# From British Associationism to the Hippocampal Cognitive Map: A Personal View From a Ringside Seat at the Cognitive/PDP Revolution

**DOI:** 10.1002/hipo.23662

**Published:** 2024-12-12

**Authors:** Patricia E. Sharp

**Affiliations:** ^1^ Department of Psychology Bowling Green State University Bowling Green Ohio USA

**Keywords:** connectionist models, head direction cells, path integration, place cells, Rescorla–Wagner equation, subicular universal map cells

## Abstract

The mandate for this special issue of Hippocampus was to provide a few examples of one's own work in a relatively personal context. Accordingly, I will discuss some of my own work here, but will also provide a broader arc of ideas and discoveries within which the efforts of myself and many others have taken place. This history begins with the associationists, who proposed that the human mind could be understood, in part, as a compounding of simple associations between contiguously occurring items and events. This idea was taken up by the behaviorist traditions, which made significant progress toward refining this simple idea. Subsequently, as interest turned toward neural mechanisms, Donald Hebb provided a foundational proposal for how synaptic changes could provide for this associative learning. The associationist view was, however, challenged by gestaltists who took a more wholistic, cognitive approach. Stunning support was provided for Tolman's cognitive map idea with the discovery of place cells in the hippocampus, and the subsequent treatise provided in O'Keefe and Nadel's *The Hippocampus as a Cognitive Map*. I propose that, ultimately, this associationist versus cognitive debate was settled by the development of the Parallel Distributed Processing (PDP) approach, which incorporated Hebbian synapses into large, neural‐like networks, which could accomplish complex cognitive tasks. My own work took place within the framework provided by O'Keefe and Nadel. One aspect of my work followed Jim Ranck's discovery of Head Direction cells. Tad Blair and others in my lab traced a brainstem circuit, which we proposed could explain the origins of the directional code. In other work, I investigated cells in the subicular region. These provided a contrast to the hippocampal place cells in that each subicular cell kept the same spatial pattern across different environments, whereas the hippocampal cells formed a different map for each context.

## 1 Introduction

I was delighted to receive an invitation to contribute to this special issue of *Hippocampus*. Although the mandate for this was to speak primarily about one's own research contributions, I found myself inspired to also include additional personal experiences as an observer and modest participant in a truly astonishing period of scientific accomplishment.

I mention associationism in the title because this line of thought has dominated many of the subsequent efforts to understand the human mind and behavior. As reviewed elsewhere (e.g., Boring 1950; Mandelbaum 2020), this tradition may be said to have begun with Locke (1690/1975) and Hume (1738/1975), and continued on with a number of thinkers, including Mill (1829), Mill (1843/2018), and Bain (1855/2004).

These associationists observed that the mind is flooded with a stream of thought that seems to constantly flow from one item or event to another, and that the stream seems often to replicate patterns which have occurred in the external world. So, for example, if we think of (or are presented with) the word “Barack,” then we are likely to immediately think “Obama,” and then perhaps “affordable healthcare,” “Michelle,” and so forth. These workers postulated that any two objects or ideas which are frequently presented contiguously in the outer world will become associated in the mind, so that they are presented contiguously in our inner world as well. They further reasoned that it was not just simple associations between pairs of phenomena, but that even complex mental capabilities could also be built up as a result of many compounded associations.

One line of research which developed from associationism is the behaviorist tradition which was prominent in American psychology through roughly the first seven decades of the 20th century. There were several schools of behaviorism over this time, and the one I will focus on is what came to be known as the Yale‐Iowa (or Hull‐Spence) tradition, which championed the influential Stimulus–Response (S–R) theory.

The beginning of this tradition can be attributed to Pavlov, who, famously, took the study of associations out of the realm of introspection, and into the laboratory, utilizing carefully controlled, paired stimuli, in the form of conditioned (CS) and unconditioned (US) stimuli. The resulting associations were evidenced in the form of observable, quantifiable physiological responses.

## 2 S–R Theory Versus the Cognitive Map

Perhaps the most prominent of the animal learning psychologists in the S–R tradition was Kenneth Spence (1907–1967). Spence conducted his Ph.D. research under the guidance of Robert M. Yerkes at Yale University but was, perhaps, more strongly influenced by Clark L. Hull, who was also on the Yale faculty at that time (Kendler 1967).

Hull was deeply committed to making psychology into a “real science,” capable of standing side‐by‐side with the physical sciences. Essential to this was to jettison all reliance on introspection, which had dominated earlier, unsuccessful efforts. Accordingly, Hull preferred an approach in which the learning process was investigated using extremely simple paradigms, such as the T‐maze or Classical (Pavlovian) Conditioning. All stimuli and responses were strictly operationally defined, and explanations for the learning process were provided in terms of intervening variables, such as “habit strength” (the S–R bond), drive, incentive, and so forth, each of which was precisely tied to observable events. The goal was to predict the exact probabilities of each response on each learning trial on the basis of calculations using the intervening variables.

Again, as with the original associationists, the assumption was that once the basic S–R bonds were understood in these isolated situations, the insight could be used later to understand behavior in its full complexity. Importantly, this assumption was an article of faith rather than a proven fact (Kendler and Spence 1971).

Spence eventually moved to a faculty position in the Department of Psychology at the University of Iowa, in 1938. Here, he continued Hull's efforts, but his approach was somewhat more pragmatic and wide‐ranging (Kendler and Spence 1971).

The Hull‐Spence tradition came to dominate behavioral psychology for much of the 1940s through the 1960s, but it did not go unopposed. In particular, Edward C. Tolman, influenced by the Gestalt tradition, criticized S–R theory on a number of fronts (see Tolman 1948). Tolman argued that paradigms limited to examining just one isolated glandular or motor response were, in fact, missing most of what animals actually do. Rather than “helplessly responding to a succession of external stimuli” provided by the experimenter, animals approached situations with complex, purposeful, insightful, and highly structured perceptual and goal‐oriented systems. Notably, he believed that animals spontaneously developed *cognitive maps* of their environment (not requiring reinforcement, as some S–R theorists insisted), and that these maps could later be used to respond to new challenges in a flexible, insightful manner (Tolman 1948, 1959).

History has shown that this was, indeed, apt criticism, and Tolman and his colleagues provided several experimental results, which posed challenges to both the empirical and theoretical claims of the Hull‐Spence tradition.

In turn, however, the Gestalt approach was criticized on the grounds that their theoretical efforts often involved constructs which were (1) insufficiently operationally defined, (2) harkening back to introspective and anthropomorphic notions, and (3) sometimes circular in their definitions. The concept of the cognitive map seemed to provide an example of this problematic circularity. So, the fact that animals sometimes seemed to show behavior which was “insightful” and “flexible” served as evidence that they must possess a cognitive map. But, when prompted to specify the details of the cognitive map, the answer seemed to be that it was the process which enabled flexible, insightful behavior. So, the S–R theorists might insist that, given the proper theoretical framework, any claimed insightful, flexible behavior might be revealed to be predictable and fully determined.

Spence is said to have produced 74 Ph.D. graduates over the course of his career (Kendler 1967), and these graduates fanned out across the country to investigate many different aspects of conditioning processes. One such student was Jerry Weiss, who landed at Macalester College in St. Paul, Minnesota. Jerry became my undergrad mentor there, and in this role, he ensured that I had a solid foundation in the animal learning traditions, as well as in the philosophy of science, which was also an important component of the Yale‐Iowa tradition (due to the influence of the Austrian‐born philosopher, Gustav Bergman, who was also on the faculty at Iowa).

## 3 The Rescorla–Wagner Equation

Another of Spence's students was Allan Wagner, who took up a faculty position in the department of psychology at Yale in 1959. Here, he continued with research in the Spence tradition, eventually focusing in particular on classical conditioning. Down the hall from Allan was another prominent animal learning researcher, Bob Rescorla.

My own graduate career began in 1977, when I went to Yale to join Allan's laboratory. My arrival followed on the heels of publication of the stunning Rescorla–Wagner (R–W) equation (Rescorla and Wagner 1972). In a single stroke, this equation provided a unitary explanation for a vast number of experimental findings based on decades of Pavlovian conditioning research. This model also generated new predictions, many (but not all) of which, when tested, provided powerful support for the theoretical predictions of the R–W model (see Soto et al. 2023 for review).

The main insight provided by this model was that *simple contiguity* was *not* responsible for the development of associations between stimuli, as had been proposed by the original associationists. Rather, the change in associative strength for any one CS on any one occasion depended on the combined predictive power of all stimuli present on that trial. In other words, the effects of either reinforcement (US presentation) or nonreinforcement (absence of the US) were shared by all the temporally contiguous stimuli available at that moment. The magnitude of the learning depended on the difference between the joint *prediction* of the US and the *actual* US (or no‐US) presented on that trial. This difference between the predicted versus the actual US event is known as the *prediction error*.

**FIGURE 1:**
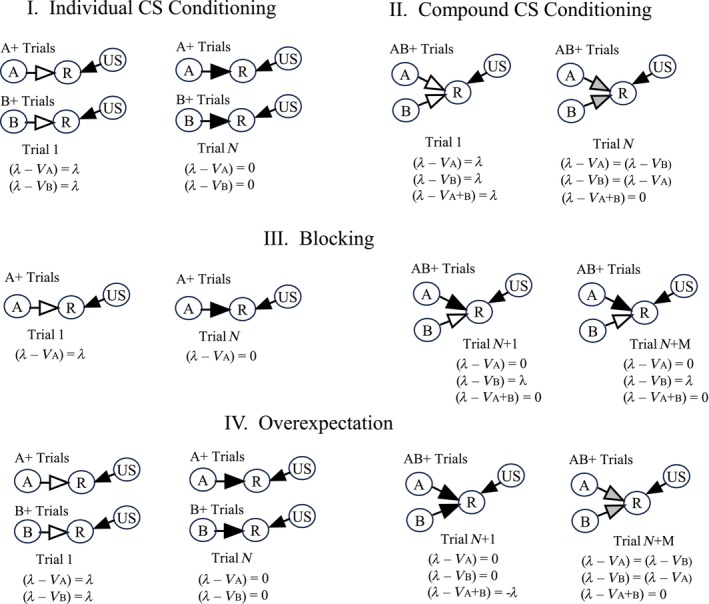
Diagrammatic representation of theoretical predictions provided by the Rescorla‐Wagner model. A and B are units which represent CS presentations. US represents US presentations. R is a response unit, presumed to initiate a behavioral response. *N* is the number of trials for each stimulus (or stimulus compound) in the first phase of an experiment. *M* is the number of trials for each stimulus (or stimulus compound) in a second phase (if applicable). Arrows show the theoretical associative strength between units, with darker arrows meant to correspond to stronger connections. The equations below each trial set indicate the theoretical prediction error for each of the stimuli or stimulus compounds at that stage of training. *λ* is the strength of conditioning afforded by the US. *V* is the current associative strength of the CS (or CS compound) on a given trial.

Figure 1 provides some examples of theoretical predictions from the R–W model. Panel I (Individual CS Conditioning) shows the most basic kind of Pavlovian conditioning trial. Here, two versions of this simple trial type are used. During A+ trials, an initially neutral stimulus (A), such as an innocuous tone or light, is used as a to‐be‐conditioned stimulus (CS). This CS is presented briefly (perhaps for 2 s) and at the end, the US (+) is presented briefly. B+ trials are identical to (and interspersed with) A+ trials, except that a different CS (e.g., tone or light) is used. The US is a stimulus, such as food or mild foot‐shock, which innately evokes a measurable response.

Note, in Figure 1, I have chosen to represent the conditioning process in the form of neuron‐like units, which are connected together, and which are equipped with synaptic plasticity. It should be noted, however, that Rescorla and Wagner were careful not to use any neural‐like language when discussing this work, because they felt it was premature at that time.

At the beginning of Trial 1 (Panel I), neither the A nor the B CS has the ability to activate the Response unit (R). This is indicated by the white color of the arrows connecting each of the CSs to the Response unit. The theoretical prediction error is calculated as the difference between the strength of association (*λ*) offered by the US versus the current strength of the association (*V*A or *V*B) between the CS and the R.

At the beginning of training, this prediction error equals *λ*, since the *V* values are assumed to be zero. At the end of some number (*N*) of trials *V*_X_ has grown to equal *λ*, and no further conditioning takes place.

These results from Panel I can be handled by an equation, which had long been used by Hull and others to describe the negatively accelerated development of conditioned associations. Specifically:

(1) $$\mathrm{\Delta VX}=\beta \left(\lambda –Vx\right)$$

Here, *V* is the current strength of the CS‐US association for a given CS, *β* is a learning rate parameter, *λ* is the asymptotic level of conditioning afforded by the US, and *V*x is the associative strength of a given CS on that trial.

Panel II shows predictions for a somewhat more complicated trial type. Here, the trials are exactly like those in Panel I, except that A and B are presented together, and this is the only type of trial. Note that, at the end of *N* trials, neither A nor B have attained the same level of associative strength as they did when presented alone in Panel I. Rather, they end up *sharing* the total *λ* value. Of special importance is that each CS has been contiguously presented with the US the same number of times as in Panel I, yet they do not reach the same level of associative strength.

Panel III illustrates the blocking phenomenon (Kamin 1968), which was one of the most important findings to prompt the development of the R–W model. Over the course of trials 1 through N, the A CS develops a strong association to the US, as in Panel I. Following this, in trials *N* + 1 through *M*, the B CS is added to the A+ presentations. The typical result for this paradigm is that the development of associative strength for the B stimulus is blocked, due to the simultaneous presence of the A stimulus, which already predicts US occurrence at the time that B is added. This blocking phenomenon again provides a demonstration that contiguity alone is not the sufficient driver for the development of associative learning. Note that Panels I, II, and III all show the same number of US pairings for the B stimulus (assuming *N* = *M*), yet the level of conditioning strength for B differs across the three.

To explain results of this kind, Rescorla and Wagner proposed a slight, but critical change to Equation (1). Specifically, they suggested that the prediction error should not be calculated for each of the CSs separately, but should be calculated just once, based on the total strength of prediction provided by all CSs presented on a given trial. Accordingly, Equation (1) was modified as shown:

(2) $$R–W\;\text{equation}:\Delta V_{X}=\beta \left(\lambda –\Sigma V_{i}\right)$$

Panel IV of Figure 1 shows one of the many findings which were predicted by this equation. Here, in trials 1 through N, A and B are each conditioned separately, to asymptote, as in Panel I. Then, compound presentations of the AB stimuli begin. Note that over the course of the AB—US pairings, the A and B stimuli actually *lose* associative strength! Thus, the A and B stimuli come to share the associative strength afforded by the US, just as they did in Panel II. The only difference is that in the overexpectation design, the A and B stimuli must lose, rather than gain strength as a result of being paired with the US.

Interestingly, although this work was done strictly at the behavioral level, the Rescorla–Wagner equation provided certain core concepts which had also appeared as foundational aspects of Parallel Distributed Processing (PDP) learning rules (see below).

## 4 New Progress for the Hebb Synapse

In 1949, Donald Hebb famously proposed a mechanism through which synaptic plasticity could be envisioned to provide a basis for associative memory. The core idea is given in his famous book *The Organization of Behavior* (Hebb 1949):

Let us assume that the persistence or repetition of a reverberatory activity (or "trace") tends to induce lasting cellular changes that add to its stability. … When an axon of cell A is near enough to excite a cell B and repeatedly or persistently takes part in firing it, some growth process or metabolic change takes place in one or both cells such that A's efficiency, as one of the cells firing B, is increased.

**FIGURE 2:**
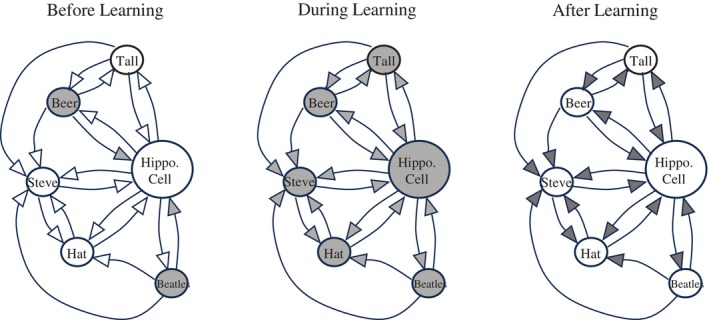
Associative learning in a Hebbian cell assembly. In each of the three diagrams, the set of smaller units (arranged in a semicircle) represent cortical cells, which send axons to project across the cell layers in the hippocampus. The larger, more centrally located unit represents a single hippocampal cell, which happens to receive input from each of the depicted cortical cells. Synaptic connections between cells are indicated by arrows. Left panel: an individual is sitting in a bar and certain cortical cells are activated (shown by gray shading) by the sensory input from the beer which the individual is drinking and from the Beatles tunes playing in the background. These two inputs alone are not strong enough at this point to activate the hippocampal cell. Middle panel: a man named Steve enters the bar and is introduced to our individual. Steve is wearing a distinctive hat and is tall. These attributes of Steve activate some additional cortical cells, and this is sufficient to activate the hippocampal cell. Then, due to the feedback connections from the hippocampus, and lateral connections among the cortical cells, every cell in the assembly becomes active. According to Hebb's rule, all involved synapses become stronger, due to coactivity in the many pre‐ and postsynaptic pairs. Right panel: following the episode at the bar the synapses remain strong, so that reinstatement of only a few of the original inputs can reactivate the entire assembly.

An illustration of how Hebb's mechanism could orchestrate associative memory in a postulated cortico‐hippocampal circuit is provided in Figure 2. Imagine an individual sitting in her favorite bar and grill, drinking a beer, with Beatles tunes playing in the background. Some friends walk in, and they introduce her to a man named Steve, who is tall and is wearing a blue baseball cap. Particular cortical cells code each of these stimuli and randomly pick the illustrated hippocampal cell to fire. Reciprocal connections back to, as well as between the cortical cells will all be active during this episode, thus repeatedly helping each other to fire. Eventually, the connections between the co‐active cells will strengthen, in accordance with Hebb's rule. At a later date, reinstatement of a smaller subset of the original stimuli may reactivate the entire assembly, thus providing content‐addressable associative episodic memory. Thus, if later our individual sees a tall man in a blue baseball cap on the street, she may be able to call him by name.

I should note that this scheme (Figure 2) for the hippocampal role in episodic memory is based on the seminal work of Teyler and DiScenna (1986) as well as on further developments by Teyler and Rudy (2007); see Rudy (2024) for a review of these ideas.

Note, the diagrams in Figure 1 include a similar (Hebbian) logic as those from Figure 2. In both cases, joint activation of a pre and postsynaptic element is required to cause an increase in synaptic strength at that synapse. One difference, though, is that in Figure 1 the presence of the US serves as a “teaching input” which, prior to any learning, is innately capable of firing the postsynaptic cell (R) on its own. For the circuit in Figure 2, there is no such teaching unit; instead, a given postsynaptic cell is chosen to fire through the joint action of numerous inputs, none of which can fire the postsynaptic cell on its own. A second difference is that, to my knowledge, Hebb did not recognize the need for presynaptic inputs to share the strength of associative changes, as was later prescribed by the R–W equation.

Hebb's postulate took on tremendous importance due to its intuitive appeal. However, at the time of Hebb's writing there were no apparent means to test for the proposed logic in a real nervous system.

That began to change as workers such as Per Andersen began to develop the ability to stimulate and record from populations of cells in the live mammalian brain. In particular, Bliss and Lømo (1973) and Bliss and Gardner‐Medwin (1973), working in Andersen's laboratory, provided stunning demonstrations that repeated, high frequency stimulation of the perforant path afferent fibers from the entorhinal cortex to the hippocampal dentate gyrus cells could result in an increase in the synaptic connection between the two cell populations (see Bliss (2024) and Lomo (2024) in this issue for reviews of the heroism involved in this discovery). This increase came to be known as long‐term potentiation (LTP). Importantly, this change was seen to last for many hours following the treatment, so that it seemed to be a viable candidate mechanism for long‐term memory storage. This was a major step toward proving that Hebb's postulate could be correct as a basis for associative learning.

However, these initial studies did not provide evidence for the core of Hebb's postulate: the synaptic plasticity *requires* that the presynaptic cell repeatedly *help to fire* the postsynaptic cell. This proof was provided several years later in a paper by McNaughton, Douglas, and Goddard (1978). They utilized the fact that the medial and lateral components of the perforant path projection could be stimulated separately, to activate two, presumably largely nonoverlapping, sets of axons. It was known, however, that these axons connected with highly overlapping sets of dentate cells. These workers carefully adjusted the intensity of stimulation for each of the lateral and medial perforant path electrodes so that neither electrode caused dentate cell firing on its own. It turned out that high frequency stimulation (of the sort used to induce LTP) also did not produce LTP when given to either of the component pathways on its own. However, when the high frequency stimulation was delivered on both components at once, so that the dentate cells were driven to fire, then robust and lasting LTP was seen on each of the individual pathways when tested alone.

This provided definitive proof of Hebb's core idea and was followed by an avalanche of further demonstrations of this associativity, as well as discovery of biochemical mechanisms for LTP.

As an aside, it is worth noting that considerable progress has been made in the effort to experimentally demonstrate the existence of experience‐dependent Hebbian cell assemblies like those depicted in Figure 2 (see reviews of this progress in McNaughton (2024) and Redish (2024)).

## 5 The End of the Animal Learning Tradition

Although publication of the Rescorla–Wagner model marked a triumph for the S–R tradition, it also fairly well coincided with the near total demise of that tradition, at least as it then existed. People such as Tolman from the Gestalt tradition, as well as a new breed of cognitive psychologists, had grown restless with behavioral studies which were limited to just simple S–R paradigms. In addition, new methods for investigating the physiology and neuroanatomy of the brain were making it increasingly possible to go directly into the brain to observe neural mechanisms of behavior.

Interestingly, even some of the leaders of the S–R tradition at the time seemed to recognize that the field had become so obsessed with minutia that they had lost perspective. In the introduction to an important volume on classical conditioning at the time (in fact, the very volume in which the R–W model was first presented), the editors opined:

“With the advent of behaviorism, and the correlated emphasis on being “scientific”, the original objective was, to some extent, lost. Among many researchers, interest focused more and more narrowly on the parametric analysis of the data in order to better understand what was viewed as a simple form of learning, the formation of a connection between the stimulus and the response. The label “classical conditioning” came to refer more to this assumed kind of learning than to a method through which higher mental processes could be observed” (Black and Prokasy 1972).

## 6 Hebb Synapses and the Cognitive Map in the Department of Psychology at Boulder

Due to the new intellectual and technological developments, many of us working in the animal learning tradition felt the need to shift to a more biological and/or cognitive approach. For me, for a variety of reasons, this meant not only shifting my area of interest, but also moving to a different institution.

Accordingly, I left Yale, and made the move to the University of Colorado in 1979, initially planning to work with Eva Fifkova, looking for morphological changes in synaptic spines, which might be mechanisms for learning in the hippocampus. In particular, Eva and colleagues had shown that perforant path stimulation like that used to induce LTP (see above) also caused changes in spine morphology in dentate granule cells (Fifková and Van Harreveld 1977; Fifková and Anderson 1977). She advanced theoretical reasons to suggest that the observed changes might result in increases in synaptic strength like those proposed by Hebb, and observed in LTP paradigms. My effort in Eva's laboratory was an attempt to show that associative learning, at the behavioral level, could also invoke similar changes in spine morphology. As it turned out, our attempts at this came to nothing, since we observed no differences between spines in the “learning” versus the “control” group. As far as I know, changes in spine morphology are still candidates for at least some aspects of plasticity underlying associative learning, although this issue is still not fully resolved (Chidambaram 2019; Segal 2005).

Nonetheless, it turned out to be a very exciting time in the department during the 80s.

### 6.1 The Cognitive Map in the Hippocampus

Shortly after my arrival in Colorado, there emerged a buzz about the new book, *The Hippocampus as a Cognitive Map*, by O'Keefe and Nadel (1978). I have a vague memory of Eva and I together excitedly filling out the paperwork necessary to order this book. Upon its arrival, I set about the effort to master its wide‐ranging topics, and the new version of Cognitive Map theory, which harkened back to Tolman. See Nadel (2024) for a personal story of how this book came to be written.

A centerpiece of the book was the newly discovered hippocampal place cells (O'Keefe 1976; O'Keefe and Dostrovsky 1971). These remarkable cells had the property that each cell fired only when the rat was located in a particular region of the test maze. These cells were not simply driven by locally available sensory stimuli, since the place‐specific activity could withstand removal of subsets of these landmarks. O'Keefe and Nadel claimed that these place cells may constitute the innately‐given, flexible, insightful cognitive map originally postulated by Tolman. As is well known, this book provided a prominent theory of hippocampal function and it initiated a new area of study for generations of researchers.

### 6.2 Barnes and McNaughton Arrive in Boulder

To my great good fortune, just as I was devouring the O'Keefe and Nadel book, two newly minted faculty arrived in the department. These were Carol Barnes and Bruce McNaughton, and, as I recall, they were just arriving from Europe, where they had completed back‐to‐back postdoctoral studies with Per Andersen and John O'Keefe. They were, of course, well on their way to becoming two of the most important workers of their generation in hippocampal function. I became the first graduate student to join their laboratory.

During these years the laboratory produced a series of powerful results which built on and extended Bruce's work on the associativity of Hebbian plasticity (see above). This included work on the functional circuitry of the hippocampus and how this interacts with Hebbian plasticity, aging, and environmental enrichment. These studies provided important support for the idea of Hebbian LTP as the basis for learning and memory.

The first time I met John O'Keefe was, I believe, during the first year after Bruce and Carol's arrival in Colorado. They were kind enough to invite me to dinner at their house during a visit from John. In an overabundance of youthful arrogance, I launched into an attack of certain aspects of the Cognitive Map book. I do not remember the details, but I believe I was taking up the Hull‐Spence objections to vagueness and circular reasoning, which I felt the O'Keefe and Nadel book had retained in relation to the Cognitive Map construct. As I recall, John was gracefully tolerant.

### 6.3 The PDP Books and the Rescorla–Wagner Rules

Another major development was publication of the two‐volume set on PDP (Rumelhart, McClelland, and The PDP Research Group 1986; McClelland, Rumelhart, and The PDP Research Group 1986), which came from the joint efforts of researchers at several institutions, who were collectively known as the PDP research group (see Redish (2024) for further description of this group). It is difficult to overestimate the impact that these volumes had on the field of neuroscience. They provided an accessible roadmap to ideas from earlier workers, such as Marr, Sutton and Barto, Grossberg, Widrow‐Hoff and others, as well as efforts ongoing in their own PDP research group. It is not possible to explain these ideas here, but basically, this framework allowed for insights into how Hebbian mechanisms would work if enacted in multiple layers, each consisting of many cells, all acting simultaneously. The effect seemed almost magical, because it was now possible to go beyond just thinking about the Hebb synapse in isolation, with only some vague faith that this idea would somehow cohere into a full explanation of complex behavior. Instead, we now had clear algorithms for large scale neural networks based on mathematical concepts from areas such as linear algebra and probability theory. Importantly, these ideas were fully testable.

This, along with the sudden availability of personal computers, made it seem that we could all become neural network designers, with the power to easily replicate our findings and make predictions using concrete, fully specified neural‐like networks.

Clearly, Hebb's idea was central to these developments and, to my knowledge, remains central to most (or all) unsupervised learning approaches. However, as outlined by Sutton and Barto (1981), the early network modelers found that it was necessary to go beyond just the conjunctive occurrence of pre and postsynaptic firing, which Hebb had provided. It was necessary, first, to implement some kind of limit, or saturability for the strength of inputs to each postsynaptic unit and, second, to provide for *sharing* of strength among those inputs, which *reliably* predicted the postsynaptic output, and to do so at the expense of less reliable inputs. These requirements were remarkably similar to those discovered by Rescorla and Wagner during their work on associative learning in animals, even though the network developers had been driven only by computational requirements. For example, Sutton and Barto pointed out that the Widrow‐Hoff learning rule was mathematically identical to the R–W equation. As they note (p. 156):

“That these two models are, in fact identical is striking, since they were constructed for very different purposes. The Widrow‐Hoff rule was formulated as an algorithm to solve sets of linear equations, and its theory addresses convergence properties. Not only are stimulus context effects not discussed in this theory, but their existence is entirely incidental.”

## 7 Place Cells and Beyond at SUNY Downstate Medical Center in Brooklyn

Following graduation from the University of Colorado in 1987, I went on to do postdoctoral work in the laboratory of Bob Muller, John Kubie and Jim Ranck, at what was then known as the SUNY Downstate Medical Center. Very exciting developments were taking place in the study of spatial cell signals in this lab during that time.

First, Bob Muller had moved the hippocampal place cell investigations into a high‐walled open field arena into which small food pellets were occasionally dropped to random locations, so that the rats were in constant motion chasing these treats (Muller, Kubie, and Ranck 1987; see Kubie (2024) for a detailed description of the methodology used in the lab at that time). For many purposes, this was a great improvement over the maze structures in which these cells had previously been investigated. This open arena provided a stunning new vision of the shape and extent of the place cell fields. It also seemed to facilitate further investigation of the sensory and computational abilities of spatial cells.

In my work at SUNY Downstate, we provided further demonstration (expanding the findings from the O'Keefe laboratory) of the ways in which external landmark stimuli and self‐motion cues combine to shape the location‐tracking abilities of the place cells (Sharp, Kubie, and Muller 1990). In particular, we recorded place cells in a slightly modified version of the Muller style cylindrical chamber. In this version, the chamber was equipped with two visually identical white cue cards located 180° apart. This created two sets of visually identical views (180° opposite) throughout the chamber. We found that most place cells in this condition continued to show the same, single place field as it had in the previous recording sessions, when only one card was present. However, the choice of location flipped between two 180° opposite positions from one session to the next, and the choice between these two possibilities depended on the entry position for that session. When the animal's entry into the chamber was the standard spot, used during single card sessions, the field was also in its standard location. However, when the entry point was shifted by 180°, the field also shifted to maintain a constant relationship to the entry location. This indicated that the cells must use some kind of path integration mechanism to maintain a distinction between the two visually identical locations.

Second, Jim Ranck had just discovered head direction (HD) cells in the postsubicular region of the hippocampal formation (Ranck 1984; Taube, Muller, and Ranck 1990). In doing so, he had not only discovered an astonishing new type of navigation‐related cell but had also led the way out of the hippocampus to anatomically connected regions. This latter move was of tremendous importance, since cells with fascinating spatial firing properties have since been discovered throughout much of the hippocampal formation and beyond.

These HD cells have the property that each cell fires only when a freely moving animal faces in one particular direction. The shape of the directional tuning curve is roughly triangular or Gaussian over an approximately 90° range, and each cell has its own preferred firing direction (see below).

As discussed further below, the HD cells (similarly to the place cells) seem to rely on a combination of landmark information and integration of angular head velocity to track direction. Salient environmental landmarks can anchor the preferred direction for each HD cell, but the cells can also use angular path integration to help track heading when the external landmarks are either missing or ambiguous. See Sharp, Blair, and Cho (2001a) for review.

## 8 The Neuroscience of Learning and Memory at Yale

For my first faculty position, I went back to the Psychology Department at Yale in 1990 and moved into a lab just one floor up from my old mentor, Allan Wagner. Having, himself, seen the need to move toward a more biological/cognitive approach, Allan had assembled a group of neuroscience faculty interested in the neurobiology of learning and memory, and I joined this group. Allan himself had also moved to a more neural‐oriented mode of theory building (e.g., Wagner and Brandon 1989).

### 8.1 Universal Map Cells in the Subiculum

In my own research at Yale, I pursued additional work on the various cell types involved in spatial cognition. One contribution was the discovery of a new type of location‐signaling cell in the subicular region of the hippocampal formation (Sharp and Green 1994). These cells tended to fire over larger regions of the arena than the hippocampal place cells, but, nonetheless, within these large regions of activity, they showed distinctive patterns, often having one or more circular or oval hot spots.

Interestingly, these cells also showed modulation by head direction and running speed. Cells with these combined influences of location, direction, and motion have been postulated as critical elements in neural network models of path integration (e.g., McNaughton et al. 1996; Samsonovich and McNaughton 1997).

**FIGURE 3:**
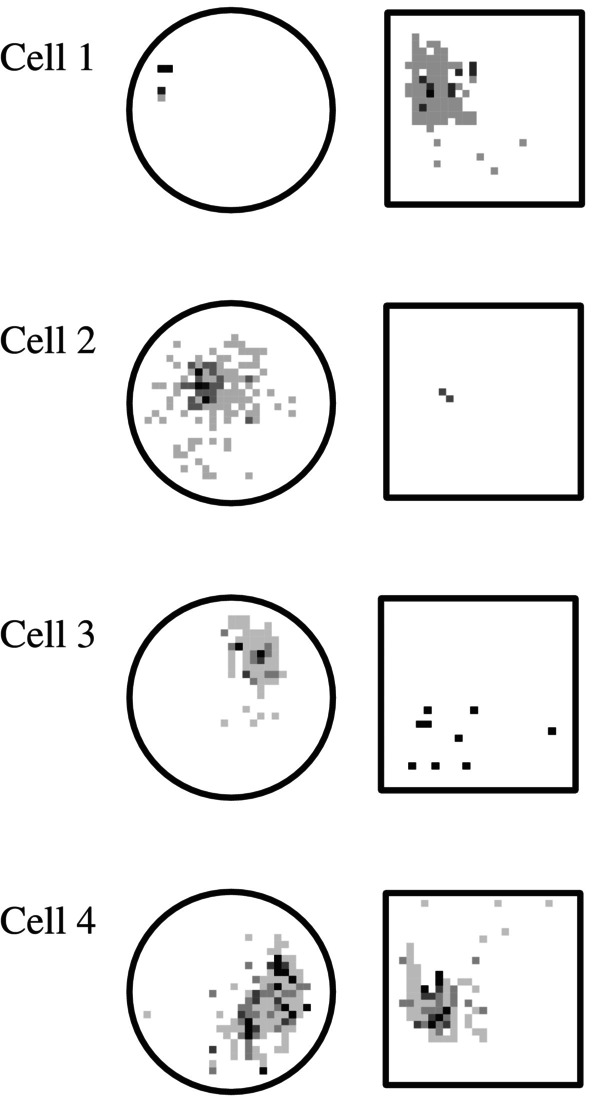
Examples of hippocampal place cells recorded in each of two visually and geometrically distinct chambers. Each of these firing rate maps shows the average rate for a cell as a function of the rat's location within a high‐walled arena of the sort pioneered by Muller and colleagues. Darker shades indicate higher rates, and no shading indicates the cell never fired when the rat was in that spot. During the 20–40 min recording sessions rats performed a pellet‐chasing task during which they continuously locomoted through the chamber, using a series of apparently random trajectories. Each of these cells shows a different spatial pattern for the square versus cylindrical chambers (from Sharp 2002).

**FIGURE 4:**
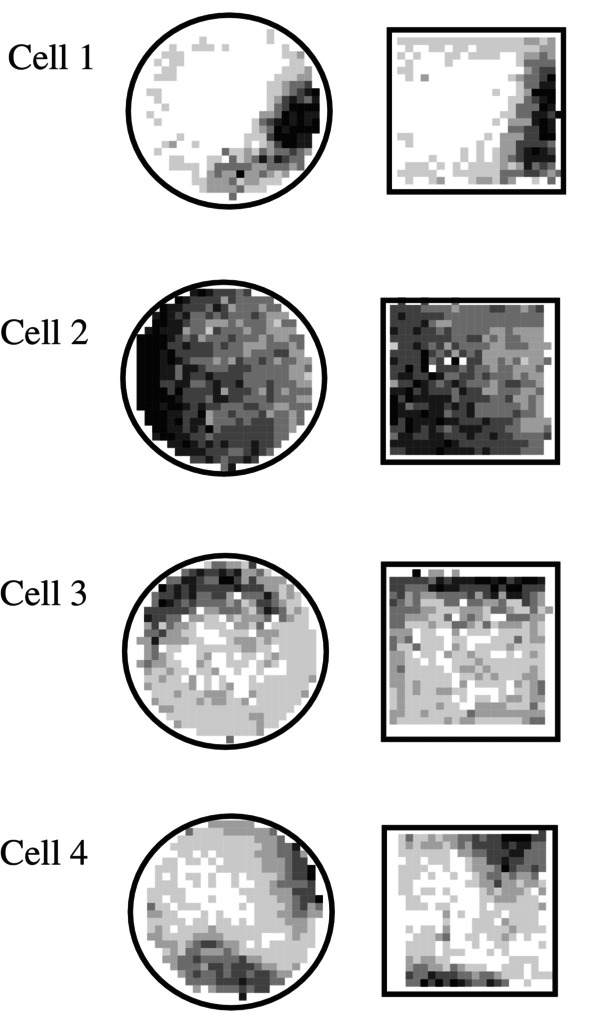
Examples of subicular cells recorded in each of two visually and geometrically distinct chambers. Firing rate maps as in Figure 3. Each of these cells shows a similar spatial pattern for the square and cylindrical chambers (from Sharp 2002).

**FIGURE 5:**
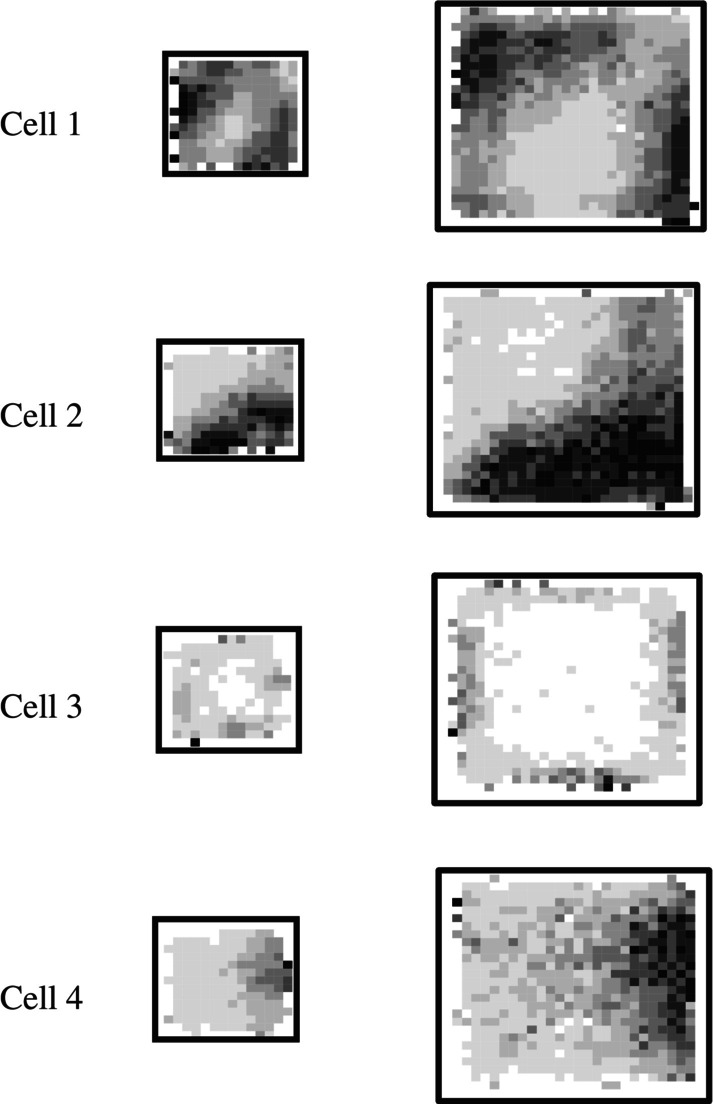
Examples of subicular cells recorded in each of a large and small chamber. Firing rate maps as in Figure 3. Note that for each cell the spatial firing pattern in the large square appears to be an expanded version of the pattern in the small square (from Sharp 2002).

An important way in which these subicular signals were fundamentally different from those of hippocampal place cells, was that they seemed to retain the same map across different environments, which varied in shape and size (Sharp 1997, 1999b; Figures 3, 4, 5). The pattern for each cell appeared to morph to fit into each new environment.

This led to the suggestion (Sharp 1999a) that the hippocampus and the subiculum might play different, but complimentary roles in spatial coding. For example, animals have the ability to venture out from an initial home base in search of food, water, etc. Upon locating this goal, they can then return home, even in the absence of familiar or reliable environmental landmarks. This path integration ability could possibly be provided by the generic subicular maps. In contrast, any learning which requires information about events and goals which are specific to a given environment would require a map like that provided by hippocampal place cells.

### 8.2 A Postulated Brainstem Origin for Head Direction Cells

As mentioned above, HD cells were originally discovered in the postsubiculum, but have since been found in additional cortical regions, including retrosplenial cortex (Chen et al. 1994; Cho and Sharp 2001). Interestingly, these areas have strong reciprocal connections with the anterior thalamic nuclei (ATN). This connection to a specific thalamic area suggested that the HD signal might be similar to signals in other sensory modalities, such as the visual and auditory systems.

**FIGURE 6:**
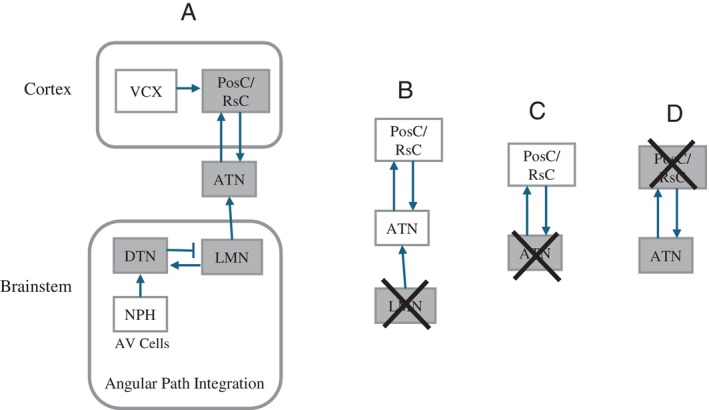
(A) Functional anatomy of the HD system. Shaded boxes represent regions that are known to contain head direction cells. (B) Lesions of the LMN abolish HD signals in “downstream” (cortical and thalamic) regions. (C) Lesions of the ATN abolish the HD signal in cortical areas. (D) Lesions of postsubiculum leave ATN HD signals largely intact. Abbreviations: ATN, anterior thalamic nucleus; DTN, dorsal tegmental nucleus; LMN, lateral mammillary nucleus; NPH, nucleus prepositus hypoglossi; PosC, postsubicular cortex; RsC, retrosplenial cortex; VCX, visual cortex.

Specifically, it seemed that the HD signal might begin in an area closer to the peripheral sensory input necessary for directional computations, and then be relayed up to cortical areas via the thalamus. One relevant type of sensory information is angular head velocity (AV), which signals changes in directional heading, and thus may be critical for an angular path integration mechanism for the HD cells. A likely pathway for providing vestibular AV input to the HD system is shown in Figure 6A, which is based on numerous anatomical studies, as reviewed elsewhere (Sharp, Blair, and Cho 2001a; Blair and Sharp 2002).

Tad Blair, working as a graduate student in my laboratory, took up the challenge of investigating this circuit. Beginning with the ATN he did, indeed, find powerful HD signals (Blair and Sharp 1995), thus supporting the hypothesis that this signal does not necessarily originate in the cortex. In addition, through an amazing feat of careful observation, Tad had noticed that the HD cells in the ATN were subtly influenced by the angular turning motion of the rat's head. In particular, if the animal happened to enter the preferred direction for a given HD cell by turning right, the cell seemed to anticipate arrival into that direction, so that it showed an anticipatory rate increase slightly to the left of the overall preferred direction. Thus, the tuning curve apparently shifted slightly to the left during a rightward turn, and the converse was true for leftward turns.

I still remember the moment when I walked into the lab and saw Tad at the computer screen with beautiful directional tuning curves of what appeared to be two pristinely well‐formed HD cells, centered on the middle of the directional firing range, but ever so slightly offset from each other. As I recall, Tad had chosen two lovely, slightly contrasting colors for the two functions (perhaps purple and lavender). The screen was like a mystical vision of HD cell perfection (in stereo). I asked him to explain.

What he had done was, first, shift the data from each of the HD cells so that they were all centered on a single direction and then could be combined to form a single plot. He then divided the data from each of the cells into action potentials, which occurred when the rat happened to be turning its head clockwise versus those during counterclockwise turns. The result was the screen plot described just above.

**FIGURE 7:**
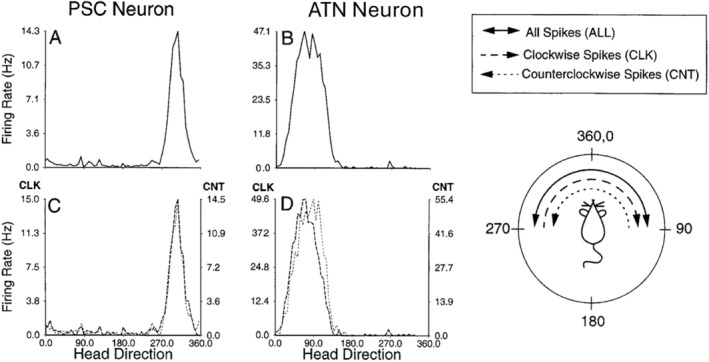
Comparison of postsubicular and anterior thalamic head direction cells. Tuning curves for a typical postsubicular (PSC) neuron (A, C) and a typical anterior thalamic (ATN) neuron (B, D) recorded during sessions in which the rat performed a pellet‐chasing task in the Muller style chamber. (A, B) Average firing rate as a function of head direction, for all samples taken over the course of the recording session. Both the PSC cell (A) and the ATN cell (B) show a distinct peak of activity when the animal faces in a specific direction. (C, D) Average firing rate as a function of direction broken down into samples, which were taken during clockwise (thick dashed line) and counterclockwise (thin dashed line) turns. The PSC cell maintains the same directional peak regardless of which way the head is turning (C). However, for the ATN cell, the directional peak during clockwise head turns is displaced to the left, and the directional peak during counterclockwise head turns is displaced to the right (D). Thus, ATN cells appear to shift their optimal firing direction as a function of angular head velocity (from Blair and Sharp 1995).

We no longer have this original diagram, but Figure 7 (Blair and Sharp 1995) shows an example of clockwise versus counterclockwise directional tuning for a single ATN cell, along with a postsubicular cell, for comparison. Interestingly, Redish (2024) has also included an anecdotal description of his first contact with these data, when he met up with Tad at a conference. This data provided support for aspects of ongoing theoretical work by Redish, Touretzky, Elga, and others (see below).

Upon further analysis, what this shifting implied was that the HD cells in ATN (but not postsubiculum) were anticipating the future directional heading of the animal by about a 20 ms interval. This is compatible with the idea that the HD signal is computed upstream of cortical regions (i.e., in brainstem regions) and then transferred to the cortex via the ATN with about a 20 ms latency.

The next step in the anatomical circuit (Figure 6A) was the lateral mammillary nucleus (LMN). Here, again, there were abundant, robust HD cell firing patterns (Blair, Cho, and Sharp 1998). These cells were also (as in the ATN) influenced by the rat's momentary angular head velocity, but here the effects were more complex. Of particular relevance here, these cells seemed to anticipate future directional heading by an even larger interval (approximately 35–40 ms) than the HD cells in ATN.

As a final step in the circuit, Sharp, Tinkelman, and Cho (2001) discovered that the dorsal tegmental nucleus (DTN) also contains HD cells, although these were significantly broader and less well defined than HD cells in other areas. In addition, there was an abundance of cells which coded for AV, as well as cells which were jointly influenced by both AV and HD. The AV cells fire at a fixed rate when the animal is still, but sharply increase their rates as a linear function of angular speed in one direction (clockwise or counterclockwise), and similarly decrease sharply for head turns in the opposite direction. Importantly, the DTN receives direct, powerful input from the vestibular system (Liu, Chang, and Wickern 1984).

These and other results enabled us to develop an informal model, based on earlier efforts (McNaughton, Chen, and Marcus 1991; Skaggs et al. 1994; Redish, Elga, and Touretzky 1996; Sharp, Blair, and Brown 1996; Zhang 1996; Goodridge and Touretzky 2000) in which the HD cell signal is initially formed in the LMN—DTN complex, and then relayed to cortical areas, where it is combined with environmental landmark information (Sharp, Blair, and Cho 2001a; Blair and Sharp 2002). This idea was supported by two main lines of evidence: (1) HD cell models predicted the basic cell properties seen in the ATN‐DTN complex and (2) lesion studies showed that LMN lesions abolish HD signals in the ATN (Blair, Cho, and Sharp 1998, 1999) and cortex (Sharp and Koester 2008), while ATN lesions abolish HD signals in the postsubiculum, but the reverse in not true (Goodridge and Taube 1997). These lesion results are summarized in Figure 6B–D.

## 9 The S–R Bond Versus Cognitive Map Controversy Has Dissolved

Since the initial discovery of the Rescorla–Wagner rules for associative learning, these same rules have been found to hold in numerous species, including many mammals and birds, as well as arthropods, flatworms, and mollusks (Soto et al. 2023). In addition, the R–W equation has been incorporated centrally into explanations for many cognitive processes, including generalization, object learning, language learning, and many more (Soto et al. 2023), as well as in many connectionist networks, as described above. The original paper on the R–W equation (Rescorla and Wagner 1972) has been cited at least 4500 times since its publication. Thus, it seems that by using such a simple learning preparation, the classical conditioning studies revealed fundamental truths, which stretch across species, tasks, and theories.

Since the discovery of hippocampal place cells and the accompanying hippocampal cognitive map theory, numerous astonishing spatial cell types, including the remarkable entorhinal grid cells (Hafting et al. 2005), have been discovered throughout the hippocampal formation and related limbic and brainstem areas (see above). This would seem to vindicate Tolman's original speculation about an innately given, spontaneously developing, cognitive map.

So, it seems that the old debate between the S–R behaviorists and the cognitive map gestaltists has melted away in the face of a more advanced neural network understanding. In the end, the R–W associative rules have come to serve complex cognitive structures, and the cognitive maps are now explained as consisting of connectionist associations. In fact, by now, further developments based on these same connectionist concepts have made it so that the reader cannot be certain whether this essay was written by the named author, or by an AI bot.

## Data Availability

No new data were generated for this manuscript.

## References

[hipo23662-bib-0001] Bain, A. 1855/2004. The Senses and the Intellect, edited by J. W. Parker . London: Kessinger Publishing.

[hipo23662-bib-0002] Black, A. H. , and W. F. Prokasy . 1972. “Introduction.” In Classical Conditioning II: Current Theory and Research, edited by A. H. Black and W. F. Prokasy . New York: Appleton‐Century_Crofts.

[hipo23662-bib-0003] Blair, H. T. , J. Cho , and P. E. Sharp . 1998. “Role of the Lateral Mammillary Nucleus in the Rat Head Direction Circuit: A Combined Single‐Unit Recording and Lesion Study.” Neuron 21: 1387–1397. 10.1037/bne0000001. 9883731

[hipo23662-bib-0004] Blair, H. T. , J. Cho , and P. E. Sharp . 1999. “The Anterior Thalamic Head Direction Signal Is Abolished by Bilateral but Not Unilateral Lesions of the Lateral Mammillary Nucleus.” Journal of Neuroscience 19: 6673–6683. 10.1523/JNEUROSCI.19-15-06673.1999. 10414996 PMC6782818

[hipo23662-bib-0005] Blair, H. T. , and P. E. Sharp . 1995. “Anticipatory Head Direction Signals in the Anterior Thalamus: Evidence for a Thalamocortical Circuit That Integrates Angular Head Velocity to Compute Head Direction.” Journal of Neuroscience 15: 6260–6270. 10.1523/JNEUROSCI.15-09-06260.1995. 7666208 PMC6577663

[hipo23662-bib-0006] Blair, H. T. , and P. E. Sharp . 2002. “Functional Organization of the Rat Head‐Direction Circuit.” In The Neural Basis of Navigation: Evidence From Single Cell Recording, edited by P. E. Sharp . Norwell, MA: Kluwer Academic Publishers.

[hipo23662-bib-0007] Bliss, T. V. P. 2024. “Pursuing Synaptic Plasticity From Cortex to LTP in the Hippocampus.” Hippocampus. This Issue. 10.1002/hipo.23665 PMC11638802 39670336

[hipo23662-bib-0008] Bliss, T. V. P. , and A. R. Gardner‐Medwin . 1973. “Long‐Lasting Potentiation of Synaptic Transmission in the Dentate Area of the Anaesthetized Rabbit Following Stimulation of the Perforant Path.” Journal of Physiology 232: 357–374. 10.1113/jphysiol.1973.sp010274. 4727085 PMC1350459

[hipo23662-bib-0009] Bliss, T. V. P. , and T. J. Lømo . 1973. “Long‐Lasting Potentiation of Synaptic Transmission in the Dentate Area of the Unanaesthetized Rabbit Following Stimulation of the Perforant Path.” Journal of Physiology 232: 331–356. 10.1113/jphysiol.1973.sp010273. 4727084 PMC1350458

[hipo23662-bib-0010] Boring, E. D. 1950. A History of Experimental Psychology. 2nd ed. New Jersey: Prentice‐Hall, Inc.

[hipo23662-bib-0011] Chen, L. L. , L. H. Lin , E. J. Green , C. A. Barnes , and B. L. McNaughton . 1994. “Head‐Direction Cells in the Rat Posterior Cortex. I. Anatomical Distribution and Behavioral Modulation.” Experimentelle Hirnforschung 101: 8–25. 7843305 10.1007/BF00243212

[hipo23662-bib-0012] Chidambaram, S. B. 2019. “Dendritic Spines: Revisiting the Physiological Role.” Progress in Neuro‐Pharmacology and Biological Phychiatry 92: 161–193. 10.1016/j.pnpbp.2019.01.005. 30654089

[hipo23662-bib-0013] Cho, J. , and P. E. Sharp . 2001. “Head Direction, Place, and Movement Correlates for Single Cells in the Rat Retrosplenial Cortex.” Behavioral Neuroscience 115: 3–25. 10.1037/0735-7044.115.1.3. 11256450

[hipo23662-bib-0014] Fifková, E. , and C. L. Anderson . 1977. “Stimulation‐Induced Changes in Dimensions of Stalks of Dendritic Spines in the Dentate Molecular Layer.” Experimental Neurology 74: 621–627. 10.1016/0014-4886(81)90197-7 7297640

[hipo23662-bib-0015] Fifková, E. , and A. Van Harreveld . 1977. “Long‐Lasting Morphological Changes in Dendritic Spines of Dentate Granular Cells Following Stimulation of the Entorhinal Area.” Journal of Neurocytology 6: 211–230. 10.1007/BF01261506. 856951

[hipo23662-bib-0016] Goodridge, J. P. , and J. S. Taube . 1997. “Interaction Between the Postsubiculum and Anterior Thalamus in the Generation of Head Direction Cell Activity.” Journal of Neuroscience 17, no. 23: 9315–9330. 10.1523/JNEUROSCI.17-23-09315.1997. 9364077 PMC6573595

[hipo23662-bib-0017] Goodridge, J. P. , and D. S. Touretzky . 2000. “Modeling Attractor Deformation in the Rodent Head‐Direction Cell System.” Journal of Neurophysiology 83: 3402–3410. 10.1152/jn.2000.83.6.3402. 10848558

[hipo23662-bib-0018] Hafting, T. , M. Fyhn , S. Molden , M. B. Moser , and E. I. Moser . 2005. “Micro‐Structure of a Spatial Map in the Entorhinal Cortex.” Nature 436: 801–806. 10.1038/nature03721. 15965463

[hipo23662-bib-0019] Hebb, D. O. 1949. The Organization of Behavior. New York: Wiley.

[hipo23662-bib-0020] Hume, D. 1738/1975. “A Treatise of Human Nature.” In A Treatise of Human Nature, edited by P. H. Nidditch . Oxford: Clarendon Press.

[hipo23662-bib-0021] Kamin, L. J. 1968. “Attention‐Like Processes in Classical Conditioning.” In Miami Symposium on the Prediction of Behavior: Aversive Stimulation, edited by M. R. Jones . Miami: University of Miami Press.

[hipo23662-bib-0022] Kendler, H. H. 1967. “Kenneth W. Spence: 1907‐1967.” Psychological Review 74: 335–341. 4864832 10.1037/h0024873

[hipo23662-bib-0023] Kendler, H. H. , and J. T. Spence . 1971. “Tenets of Neobehaviorism.” In Essays in Neobehaviorism: A Memorial Volume to Kenneth W. Spence, edited by H. H. Kendler and J. T. Spence . New York: Appleton‐Century‐Crofts.

[hipo23662-bib-0024] Kubie, J. L. 2024. “How Ideas About Context and Remapping Developed in Brooklyn.” Hippocampus. This Issue. 10.1002/hipo.23671 39727919

[hipo23662-bib-0025] Liu, R. , L. Chang , and G. Wickern . 1984. “The Dorsal Tegmental Nucleus: An Axoplasmic Transport Study.” Brain Research 310: 123–132. 10.1016/0006-8993(84)90015-5. 6434154

[hipo23662-bib-0026] Locke, J. 1690/1975. “An Essay Concerning Human Understanding.” In An Essay Concerning Human Understanding, edited by P. H. Nidditch . Oxford: Clarendon Press.

[hipo23662-bib-0027] Lomo, T. 2024. “LTP: The Accidental Discovery.” Hippocampus. This Issue.

[hipo23662-bib-0028] Mandelbaum, E. 2020. “Associationist Theories of Thought.” In The Stanford Encyclopedia of Philosophy, edited by E. N. Zalta and U. Nodelman . Stanford, California: Stanford Press. https://plato.stanford.edu/archives/win2022/entries/associational‐thought/.

[hipo23662-bib-0029] McClelland, J. L. , D. E. Rumelhart , and The PDP Research Group . 1986. “Parallel Distributed Processing: Explorations in the Microstructure of Cognition.” In Volume 1I: Psychological and Biological Models. Cambridge, MA: MIT Press. 10.7551/mitpress/5236.001.0001.

[hipo23662-bib-0030] McNaughton, B. L. 2024. “Neuronal ‘Ensemble’ Recording and the Search for the Cell Assembly: A Personal History.” Hippocampus. This Issue. 10.1002/hipo.23669 PMC11647560 39676610

[hipo23662-bib-0031] McNaughton, B. L. , C. A. Barnes , J. L. Gerrard , et al. 1996. “Deciphering the Hippocampal Polyglot: The Hippocampus as a Path Integration System.” Journal of Experimental Biology 199, no. 1: 173–185. 10.1242/jeb.199.1.173. 8576689

[hipo23662-bib-0032] McNaughton, B. L. , L. L. Chen , and E. J. Marcus . 1991. “‘Dead Reckoning’, Landmark Learning, and the Sense of Direction: A Neurophysiological and Computational Hypothesis.” Journal of Cognitive Neuroscience 3: 190–201. 10.1162/jocn.1991.3.2.190. 23972093

[hipo23662-bib-0033] McNaughton, B. L. , R. M. Douglas , and G. V. Goddard . 1978. “Synaptic Enhancement in Fascia Dentata: Cooperativity Among Coactive Afferents.” Brain Research 157: 277–293. 10.1016/0006-8993(78)90030-6. 719524

[hipo23662-bib-0034] Mill, J. 1829. Analysis of the Phenomena of the Human Mind. New Bridge Street, London: Baldwin‐Cradock.

[hipo23662-bib-0035] Mill, J. S. 1843/2018. System of Logic, Ratiocinnative and Inductive, Reprinted in System of Logic, Ratiocinnative and Inductive: A Connected View of the Principles of Evidence, and the Methods of Scientific Investigation. North Charleston, South Carolina: Hard Press.

[hipo23662-bib-0036] Muller, R. U. , J. L. Kubie , and J. B. Ranck Jr. 1987. “Spatial Firing Patterns of Hippocampal Complex Spike Cells in a Fixed Environment.” Journal of Neuroscience 7: 1935–1950. 10.1523/JNEUROSCI.07-07-01935.1987. 3612225 PMC6568929

[hipo23662-bib-0037] Nadel, L. 2024. “Some Memories of Stalking the Seahorse.” Hippocampus. This Issue. 10.1002/hipo.23656 39644129

[hipo23662-bib-0038] O'Keefe, J. 1976. “Place Cell Units in the Hippocampus of the Freely Moving Rat.” Experimental Neurology 51: 78–109. 10.1016/0014-4886(76)90055-8. 1261644

[hipo23662-bib-0039] O'Keefe, J. , and J. Dostrovsky . 1971. “The Hippocampus as a Spatial Map: Preliminary Evidence From Unit Activity in the Freely Moving Rat.” Brain Research 34: 171–175. 10.1016/0006-8993(71)90358-1. 5124915

[hipo23662-bib-0040] O'Keefe, J. , and L. Nadel . 1978. The Hippocampus as a Cognitive Map. Oxford, England: Oxford University Press.

[hipo23662-bib-0041] Ranck, J. B., Jr. 1984. “Head‐Direction Cells in the Deep Layers of the Dorsal Presubiculum in Freely Moving Rats.” Society for Neuroscience—Abstracts 10: 599.

[hipo23662-bib-0042] Redish, A. D. 2024. “Mental Time Travel: A Retrospective.” Hippocampus. This Issue. 10.1002/hipo.23661 PMC11647447 39676592

[hipo23662-bib-0043] Redish, A. D. , A. N. Elga , and D. S. Touretzky . 1996. “A Coupled Attractor Model of the Rodent Head Direction System.” Network 7: 671–686. 10.1088/0954-898X/7/4/004.

[hipo23662-bib-0044] Rescorla, R. A. , and A. R. Wagner . 1972. “A Theory of Pavlovian Conditioning: Variation in the Effectiveness of Reinforcement and Nonreinforcement.” In Classical Conditioning II: Current Research and Theory, edited by A. H. Black and W. F. Prokasy . New York: Appleton‐Century_Crofts.

[hipo23662-bib-0045] Rudy, J. W. 2024. “Memory Development, Configurations, Conjunctions, and the Hippocampal Index.” Hippocampus. This Issue. 10.1002/hipo.23658 39663644

[hipo23662-bib-0046] Rumelhart, D. E. , J. L. McClelland , and The PDP Research Group . 1986. “Parallel Distributed Processing: Explorations in the Microstructure of Cognition.” In Volume 1: Foundations. Cambridge, MA: MIT Press. 10.7551/mitpress/5236.001.0001.

[hipo23662-bib-0047] Samsonovich, A. , and B. L. McNaughton . 1997. “Path Integration and Cognitive Mapping in a Continuous Attractor Neural Network Model.” Journal of Neuroscience 17, no. 15: 5900–5920. 10.1523/JNEUROSCI.17-15-05900.1997. 9221787 PMC6573219

[hipo23662-bib-0048] Segal, M. 2005. “Dendritic Spines and Long‐Term Plasticity.” Nature Reviews Neuroscience 6: 277–284. 15803159 10.1038/nrn1649

[hipo23662-bib-0049] Sharp, P. E. 1997. “Subicular Cells Generate Similar Spatial Firing Patterns in Two Geometrically and Visually Distinctive Environments; Comparison With Hippocampal Place Cells.” Behavioral Brain Research 85: 71–92. 10.1016/S0166-4328(96)00165-9. 9095343

[hipo23662-bib-0050] Sharp, P. E. 1999a. “Complimentary Roles for Hippocampal Versus Subicular/Entorhinal Place Cells in Coding Place, Context, and Events.” Hippocampus 9: 432–443. 10.1002/(SICI)1098-1063(1999)9:4<432::AID-HIPO9>3.0.CO;2-P. 10495024

[hipo23662-bib-0051] Sharp, P. E. 1999b. “Subicular Place Cells Expand/Contract Their Spatial Firing Patterns to Fit the Size of the Environment in an Open Field, but Not in the Presence of Barriers: Comparison With Hippocampal Place Cells.” Behavioral Neuroscience 113: 643–662. 10.1037/0735-7044.113.4.643. 10495074

[hipo23662-bib-0052] Sharp, P. E. 2002. “Subicular Place Cells Show Similar Firing Fields Across Different Environments: Comparison With Hippocampal Place Cells.” In The Neural Basis of Navigation: Evidence From Single Cell Recording, edited by P. E. Sharp . Norwell, MA: Kluwer Academic Publishers.

[hipo23662-bib-0053] Sharp, P. E. , H. T. Blair , and M. A. Brown . 1996. “Neural Network Modeling of the Hippocampal Formation Signals and Their Possible Role in Navigation: A Modular Approach.” Hippocampus 6: 720–734. 10.1002/(SICI)1098-1063(1996)6:6<720::AID-HIPO14>3.0.CO;2-2. 9034858

[hipo23662-bib-0054] Sharp, P. E. , H. T. Blair , and J. Cho . 2001a. “The Anatomical and Computational Basis of the Head Direction Cell Signal in the Rat.” Trends in Neurosciences 24: 289–294. 10.1016/S0166-2236(00)01797-5. 11311382

[hipo23662-bib-0055] Sharp, P. E. , and C. Green . 1994. “Spatial Correlates of Firing Patterns of Single Cells in the Subiculum of the Freely‐Moving Rat.” Journal of Neuroscience 14: 2339–2356. 10.1523/JNEUROSCI.14-04-02339.1994. 8158272 PMC6577112

[hipo23662-bib-0056] Sharp, P. E. , and K. Koester . 2008. “Lesions of the Mammillary Body Region Severely Disrupt the Cortical Head Direction, but Not Place Cell Signal.” Hippocampus 18: 766–784. 10.1002/hipo.20436. 18446828

[hipo23662-bib-0057] Sharp, P. E. , J. L. Kubie , and R. U. Muller . 1990. “Firing Properties of Hippocampal Neurons in a Visually‐Symmetrical Environment: Contributions of Multiple Sensory Cues and Mnemonic Processes.” Journal of Neuroscience 10: 3093–3105. 10.1523/JNEUROSCI.10-09-03093.1990. 2398374 PMC6570250

[hipo23662-bib-0058] Sharp, P. E. , A. Tinkelman , and J. Cho . 2001. “Angular Velocity and Head Direction Signals Recorded From the Dorsal Tegmental Nucleus of Gudden in the Rat; Implications for Path Integration in the Head Direction Cell Circuit.” Behavioral Neuroscience 115: 571–588. 10.1037/0735-7044.115.3.571. 11439447

[hipo23662-bib-0059] Skaggs, W. , J. Knierim , H. Kudrimoti , and B. L. McNaughton . 1994. “A Model of the Neural Basis of the Rat's Sense of Direction.” In Advances in Neural Information Processing Systems, 7, edited by G. Tesauro , D. Toureszky , and T. Leen . Cambridge, Massachusetts: MIT Press. 11539168

[hipo23662-bib-0060] Soto, F. A. , E. H. Vogel , Y. Uribe‐Bahamonde , and O. D. Perez . 2023. “Why Is the Rescorla‐Wagner Model So Influential?” Neurobiology of Learning and Memory 204: 107794. 10.1016/j.nlm.2023.107794. 37473985

[hipo23662-bib-0061] Sutton, R. S. , and A. G. Barto . 1981. “Toward a Modern Theory of Adaptive Networks.” Psychological Review 88: 135–170. 7291377

[hipo23662-bib-0062] Taube, J. S. , R. U. Muller , and J. B. Ranck Jr. 1990. “Head‐Direction Cells Recorded From the Postsubiculum in Freely Moving Rats. I. Description and Quantitative Analysis.” Journal of Neuroscience 10: 420–432. 10.1523/JNEUROSCI.10-02-00420.1990. 2303851 PMC6570151

[hipo23662-bib-0063] Teyler, T. J. , and P. DiScenna . 1986. “The Hippocampal Memory Indexing Theory.” Behavioral Neuroscience 100: 147–152. 3008780 10.1037//0735-7044.100.2.147

[hipo23662-bib-0064] Teyler, T. J. , and J. W. Rudy . 2007. “The Hippocampal Indexing Theory and Episodic Memory: Updating the Index.” Hippocampus 17: 1158–1168. 17696170 10.1002/hipo.20350

[hipo23662-bib-0065] Tolman, E. C. 1948. “Cognitive Maps in Rats and Men.” Psychological Review 55: 189–208. 10.1037/h0061626. 18870876

[hipo23662-bib-0066] Tolman, E. C. 1959. “Principles of Purposive Behavior.” In Psychology: A Study of a Science, edited by S. Koch , vol. 2. New York: McGraw‐Hill.

[hipo23662-bib-0067] Wagner, A. R. , and S. E. Brandon . 1989. “Evolution of a Structured Connectionist Model of Pavlovian Conditioning (ÆSOP).” In Contemporary Learning Theories: Pavlovian Conditioning and the Status of Traditional Learning Theory, edited by S. B. Klein and R. R. Mowrer , 149–189. Hillsdale, NJ: Erlbaum.

[hipo23662-bib-0068] Zhang, K. 1996. “Representation of Spatial Orientation by the Intrinsic Dynamics of the Head Direction Cell Ensemble: A Theory.” Journal of Neuroscience 16: 2112–2126. 10.1523/JNEUROSCI.16-06-02112.1996. 8604055 PMC6578512

